# Probiotic supplements and stress‐related occupational health outcomes: A scoping review

**DOI:** 10.1002/1348-9585.12404

**Published:** 2023-05-22

**Authors:** Jin Jun, Ana Kasumova, Todd Tussing, Amy Mackos, Sheryl Justice, Jodi McDaniel

**Affiliations:** ^1^ The Ohio State University College of Nursing Columbus Ohio USA; ^2^ The Ohio State University Columbus Ohio USA

**Keywords:** occupational health, probiotics, stress, well‐being, workers

## Abstract

**Background:**

Prolonged and constant stress from work often leads to numerous adverse health effects. In recent years, interest in probiotics, living microorganisms that can benefit their host when consumed in adequate amounts, to aid health and well‐being has increased. This scoping review is to systematically evaluate the current state of science on the effects of probiotic supplements on health, stress, and stress‐related symptoms among working adults in occupational settings.

**Methods:**

We performed a systematic scoping review following the Arksey and O'Malley Framework. Studies that examined the effects of probiotics on workers' health and stress‐related indicators/outcomes in occupational settings were included. A comprehensive search was performed from November 2021 to January 2022 using MEDLINE/PubMed, Cochrane Library, CINAHL, PsychInfo, Scopus, and Embase.

**Results:**

A total of 14 papers met the inclusion and exclusion criteria. Probiotics consisted primarily of Lactobacillus and/or Bifidobacterium strains in various forms and doses. Three out of eight studies reported statistical differences in inflammatory markers or stress hormone levels between probiotic and placebo groups. Three of six reported reduced respiratory tract infection incidents in the probiotic groups and three out of four studies reported no differences in anxiety and depression between groups. Lastly, three studies found that absenteeism and presentism were lower in probiotic groups compared with placebo groups.

**Conclusion:**

The potential benefits of probiotics exist; however, the measurements of outcomes, the types of probiotics used, and the characteristics of the intervention varied across studies. Further research is needed focusing on probiotics' direct and indirect mechanisms of action on the stress response and the standardization of strains and dosing.

## 1 INTRODUCTION

Stress, a feeling of emotional or physical tension resulting from adverse or very demanding individual and environmental circumstances, is a challenging and threatening experience.^1^ Although stress is unavoidable and essential to survival, chronic stress (herein referred to as stress)—a prolonged and constant feeling of being overwhelmed over a long period^1^—is linked to a wide range of health issues from heart disease^2^, ^3^ to depression.^4^, ^5^ The idea that stress is a syndrome of the whole body has been discussed since the days of Hippocrates (460–377 BC), who identified that psychosomatic disorders were abnormal physical reactions to stressful emotions, incidents, and situations.^6^

One of the most common and persistent causes of stress for adults between 18 and 64 years old is their work,^7^ which is not surprising when an average working adult spends more than 90 000 h or one‐third of their life at work.^8^ Even though stressors at work are often external to a person (e.g., work environments and workload), stressors initiate both physiological and psychological responses that can be detrimental to an individual's health over time.^9^, ^10^, ^11^ Since the onset of COVID‐19, nearly eight out of 10 Americans report that they suffer from a lack of interest, motivation, and/or energy as a result of occupational stress and burnout.^12^ In response, the demands for innovative and efficacious interventions to manage stress and promote well‐being for working adults are increasing.^13^

**FIGURE 1:**
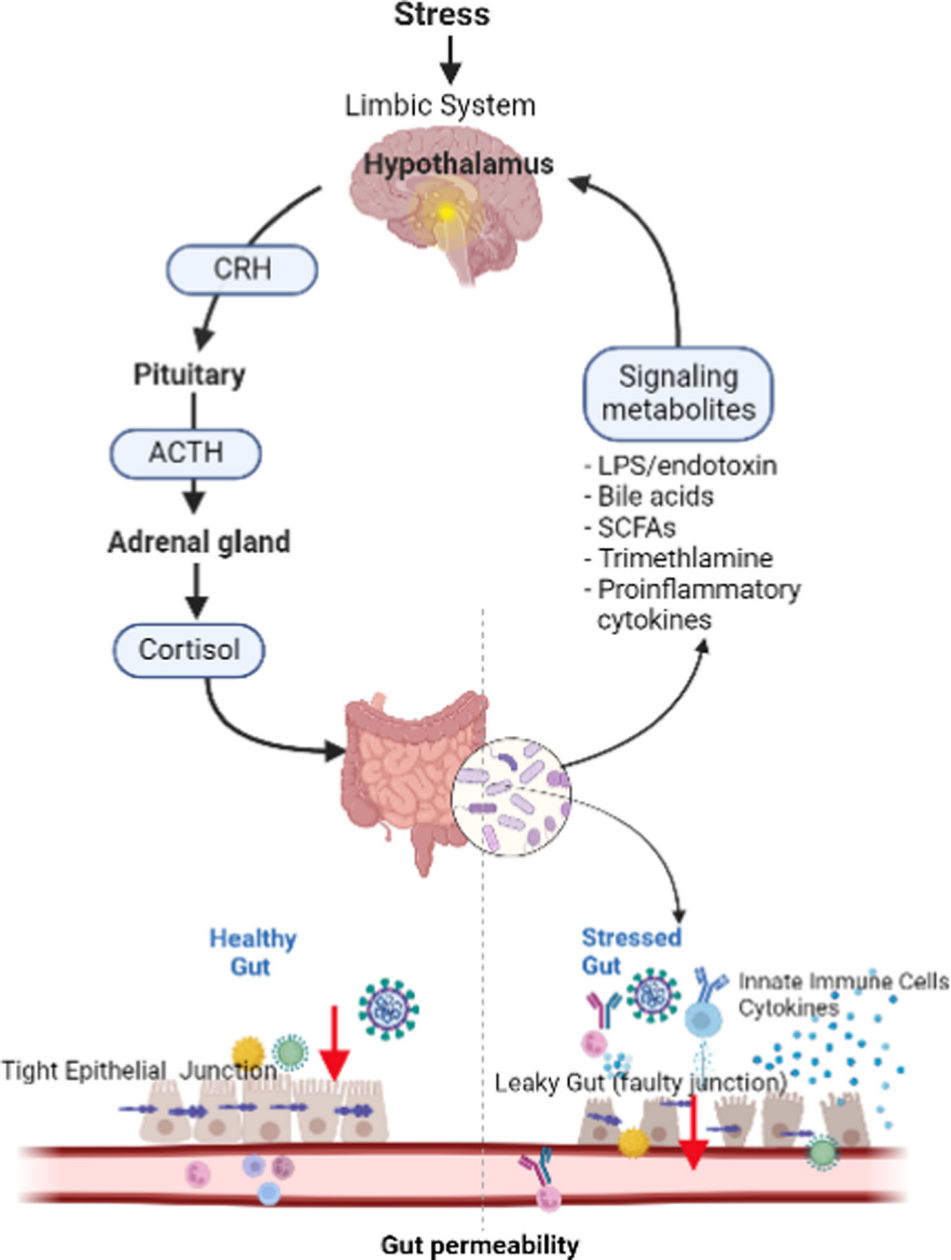
Stress and Brain–Gut Axis.

The stress response is complex, but the main drivers and regulators of the response are the stress hormones, glucocorticoids and catecholamine, which are activated through the hypothalamic–pituitary–adrenal axis and the sympathetic nervous system, and mediate the body's adaption to stress.^9^, ^14^ In particular, stress greatly affects the gut, which is considered “the second brain” due to the bidirectional communication along the gut–brain axis (GBA) through neurons and neurotransmitters found both in the gut and the brain along the autonomic, enteric, neuroendocrine, and immune system pathways.^15^, ^16^ Furthermore, chronic stress involves a cascade of inflammatory events leading to imbalances between the gut microbiome and intestinal membranes, resulting in gut inflammation and increased intestinal permeability leading to “leaky gut” (Figure 1).^17^ Accumulating evidence suggests that leaky gut is linked to alterations in behaviors and mood through the shared circuit along the GBA.^16^, ^18^

Probiotics are living microorganisms, such as *Lactobacillus sp*., *Bifidobacterium sp*., *Streptococcus thermophilus*, *and Saccharomyces boulardii*, that have been reported to confer health benefits on the host when consumed in adequate amounts by improving and restoring the gut microbiota and subsequently modulating the stress response.^19^ At an estimated annual growth rate of 7.5% and with the current global market value of over 58 billion dollars, probiotics are the fastest‐growing dietary supplement.^20^ Preclinical and some human trials have shown that probiotics decrease pathogenic gastrointestinal microorganisms and increase the synthesis of certain neurotransmitters and biologically active factors, which can potentially influence the brain via the GBA, modulating emotional responses and buffering against the harmful effects of stress.^15^, ^21^, ^22^, ^23^, ^24^, ^25^ For example, probiotics prevented bacterial translocation and improved intestinal barrier function in mice and rats following psychological stress.^15^, ^25^ In a systematic review of oxidative stress indices, such as total antioxidant capacity in human serum, probiotic supplements decreased the risk of chronic diseases by reducing circulating free radicals and increasing levels of antioxidant enzymes in the body.^26^ Furthermore, the consumption of probiotics significantly reduced subjective stress levels among healthy volunteers^27^ and self‐reported dysfunctional thoughts in depressed individuals.^28^

The use of dietary supplements to manage stress‐related health issues is typically not the focus of occupational health. However, as an increasing number of people are turning to alternative therapies to help manage their stress,^28^ it is important for occupational health clinicians to examine the role of probiotics in moderating the detrimental health effects of chronic stress in workers.^29^ Thus, the purpose of this scoping review is to systematically evaluate and determine the current state of science on the effects of probiotic supplements on health, stress, and stress‐related symptoms among working adults in occupational settings.

## 2 METHOD

The overarching objectives of scoping reviews are to identify knowledge gaps, clarify key concepts, set research agendas, and determine implications for decision‐making.^30^ Scoping reviews are the most appropriate when a certain topic is less well‐known and can serve as a precursor to a systematic review.^30^ Although different from systematic reviews, scoping reviews still require the development of an a priori protocol (PROSPERO Register: CRD42022297808) and follow the steps of pre‐defined objectives and methods of the review, which provides details of a proposed plans. For this review, we followed the Arksey and O'Malley Framework^31^ of six stages of conduct: (1) specify the research question; (2) identify relevant literature; (3) select studies; (4) map out the data; (5) summarize, synthesize, and report the results; and (6) include expert consultation through the scoping review process. We also followed the guidelines of the Preferred Reporting Items for Systematic Reviews and Meta‐Analyses (PRISMA) 2020^32^ for the design, literature search, and data extraction, synthesis and reporting.

### 2.1 Search strategy and selection criteria

In consultation with a health services librarian, literature searches were performed with keywords, MeSH terms, and Boolean operators from November 2021 through January 2022 in the following electronic databases: MEDLINE/PubMed, CINAHL, PsychInfo, Scopus, and Embase (Data S1). The search was not limited by date so as to include all relevant studies published up to the final search date in January 2022. The initial search strategy was developed in PubMed and adapted to the other search engines. Studies were included in this review if they: (1) were randomized controlled trials, (2) used intervention of oral probiotic supplementation, (3) included a sample of people in occupational settings, (4) reported measures of stress and stress‐related health indicators/outcomes, and (5) were peer‐reviewed and published in English. Studies were excluded if the study population included animals, children, or pregnant women. Gray literature (e.g., reports and working papers) and other review papers were also excluded.

### 2.2 Data management and analysis

Full citations of final search results were imported into the online citation management software Mendeley and then uploaded into the systematic review software Covidence. Two independent reviewers (JJ and AK) performed a norming exercise on Covidence to ensure consistency before reviewing titles and abstracts, excluding manuscripts that did not fit the inclusion criteria. Both reviewers also read the full text of the remaining articles to determine inclusion or exclusion in the scoping review. The reviewers resolved disagreements by discussion and used a tiebreaker as needed. Study data were extracted in Covidence using a data template developed in Extraction 1.0.

Although not required for scoping reviews, the studies included in the current review were assessed for risk of bias as they were randomized controlled trials. Critically appraising the likelihood of inaccuracies in studies estimating overall intervention effects is especially prudent when studying less well‐known topics.^30^ While several tools are available to aid with this step, we selected the Joanna Briggs Institute (JBI) Critical Appraisal Checklist for randomized controlled trials because of its ease of use and wide acceptability.^31^ There are 13 questions on the JBI Checklist for randomized controlled trials, including the random sequence generation, allocation concealment, blinding of participants and personnel, blinding of outcome assessment, incomplete outcome data, selective reporting, and other biases.^32^ Each question is answered as either yes, no, or unclear. Two independent reviewers (JJ and AK) used this tool to determine the quality of reviewed articles. The decision was made to exclude studies with poor ratings (indicating a significant risk of bias), determined when more than seven questions were answered as no. However, no studies were rated as poor. When there was a discrepancy on more than three questions between the two reviewers, a third reviewer was invited to provide an independent appraisal.

### 2.3 Data extraction

Relevant data on study design, sample characteristics, setting, and probiotic intervention, the measurable outcomes, statistical results, and implications of the findings were extracted by two authors (JJ and AK) and entered into the matrix created on Covidence software. The extracted data were then further synthesized based on the thematically grouped outcomes determined by the first author and agreed upon by the other authors. Database search results were reported following the 2020 PRISMA Guidelines.^33^

## 3 RESULTS

### 3.1 Search results

**FIGURE 2:**
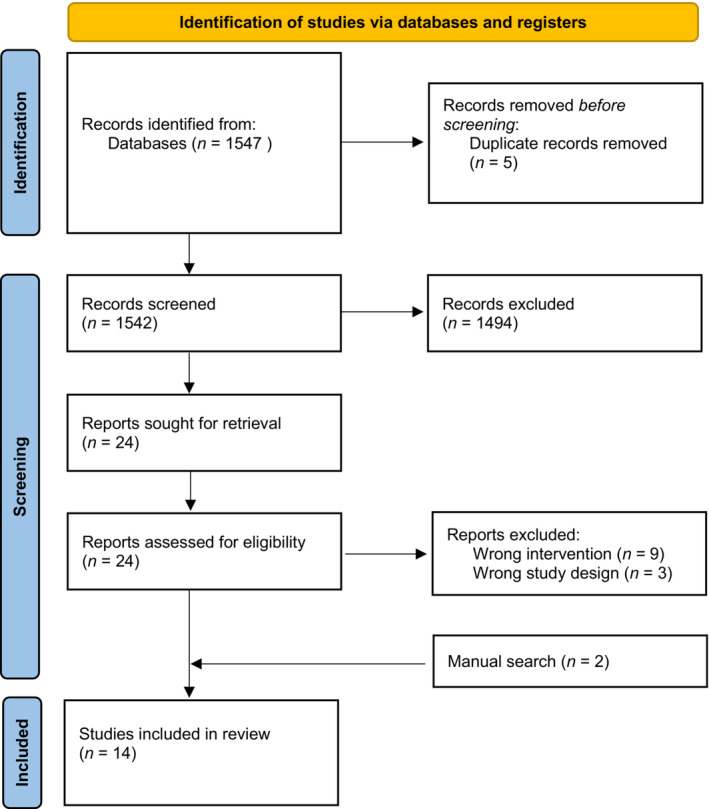
PRISMA Diagram of the Literature Search. *Source*: Page et al.^33^

The PRISMA flow diagram of the study selection process is shown in Figure 2. The initial search resulted in 1547 publications. After removing duplicates, 1542 titles and abstracts were screened and a total of 24 studies were moved forward for a full‐text review. Of these 24 studies, nine were excluded because the probiotic intervention was not delivered orally and three were excluded because they were not clinical trials. Two additional studies were identified when reviewing reference lists during the full‐text review process, resulting in 14 papers in the review.^34^, ^35^, ^36^, ^37^, ^38^, ^39^, ^40^, ^41^, ^42^, ^43^, ^44^, ^45^, ^46^, ^47^

### 3.2 Sample characteristics

The characteristics of the 14 studies included in this review are summarized in Table 1. The most common study design among the sample was the randomized double‐blinded placebo controlled trial,^34^, ^35^, ^36^, ^37^, ^38^, ^39^, ^40^ followed by open‐label randomized controlled trial,^41^, ^42^, ^43^ randomized three‐arm parallel design,^44^, ^45^, ^46^ and secondary data analysis of a randomized controlled trial.^47^ The studies were conducted worldwide. Half were implemented in Asia (China,^43^ Japan,^38^, ^41^, ^42^, ^47^ and Iran^44^, ^45^). The remainder of the studies was performed in Germany,^34^, ^37^ Finland,^35^ Italy/Albania,^36^ Sweden,^40^ the United States,^39^ and Australia.^46^ The sample sizes ranged from 33^39^ to 1000^34^ participants. In total, there were 3907 participants, with 1989 in intervention groups and 1918 in control groups. The age of the participants across all included studies ranged from 26 years to 45 years. Only half of the studies reported the sex of the participants. Of the studies that reported participants' sex, 47.6% of the participants were women. The most common occupational group studied was healthcare workers.^34^, ^36^, ^39^, ^41^, ^43^, ^46^, ^47^ followed by factory shift workers,^40^, ^44^, ^45^ office workers,^38^, ^42^ and military personnel.^35^, ^37^ One study included a mixed sample of healthcare workers and first responders working in shifts, such as nurses, police, and firefighters.^34^

**TABLE 1:** Study characteristics (*N* = 14).

Author/Year	Country	Study design	Work setting (Occupations)	Age (mean years, intervention vs. control)	Total number of participants (intervention vs. control)	Primary outcomes	Secondary outcomes
Guillemard/2010^34^	Germany	1	Healthcare workers and first responders	31.8 vs 32.5	1000 (500 vs. 500)	Respiratory and GI infection	Inflammatory markers
Kalima/2016^35^	Finland	1	Finnish Navy and army personnel	Not reported	983 (488 vs. 495)	Respiratory and GI Infection	
Kinoshita/2019^41^	Japan	2	Healthcare workers (64.7% Nurses)	39.3 vs. 39.4	961 (479 vs. 482)	Respiratory infection	Inflammatory markers
Kinoshita/2021^47^	Japan	4	Healthcare workers (64.7% Nurses)	39.3 vs. 39.4	961 (479 vs. 482)	Sleep quality	Health and mood
Kokubo/2020^42^	Japan	2	Corporate office workers	36.2 vs. 36.4	153 (80 vs. 73)	Presentism and absenteeism	Mood
Mohammadi/2015^45^	Iran	3	Petrochemical factory workers	32.vs. 32.8	35 (10 vs. 12 vs. 13)	Stress markers	
Mohammadi/2016^44^	Iran	3	Petrochemical factory workers	33.1 vs. 33.2 vs. 31.5	70 (25 vs. 25 vs. 20)	General health and mood	Stress markers
Pacifici/2020^36^	Italy and Albania	1	Oral surgeons	Range: 26–45	40 (20 vs. 20)	Stress markers	Cardiovascular risks
Schlagenhauf/2020^37^	Germany	1	German navy personnel	27.3 vs. 26.8	72 (36 vs. 36)	Periodontal health	
Shida/2017^38^	Japan	1	Corporate office workers	40.5 vs. 40.6	99 (49 vs. 50)	Respiratory infection	Inflammatory and stress markers
Smith‐Ryan/2019^39^	United States	1	Healthcare workers (72.7% Nurses)	30.5 vs. 30.2	33 (15. vs 18)	Body composition and mood	Metabolic markers
Tubelius/2005^40^	Sweden	1	Factory workers	44 vs. 44	181 (94 vs. 87)	Absenteeism	
Wang/2021^43^	China	2	Healthcare workers (42% Nurses)	36.1 vs. 35.7	193 (98 vs. 95)	Respiratory infection	Absenteeism
West/2020^46^	Australia	3	Healthcare workers	41.8 vs. 40.7 vs. 41.6	87 (29 vs. 29 vs. 29)	Stress markers	Sleep quality

*Note*. Study design: 1—randomized double‐blinded placebo controlled trial, 2—open‐label randomized controlled trial, 3—randomized, blinded three‐arm parallel design, and 4—secondary analysis of randomized controlled trial.

### 3.3 Probiotic characteristics

The probiotic intervention varied in microorganism content, dose, and dose frequency across studies, as summarized in Table 2. All studies except one^43^ used *Lactobacillus* species as the main core genera of the microbial organism in their probiotics. More than half of the studies used multistrain probiotics consisting of various combinations of *Lactobacillus*, *Bifidobacterium*, and others.^35^, ^36^, ^39^, ^41^, ^44^, ^45^, ^46^, ^47^ However, there was no consensus regarding the combinations of organisms in the probiotic interventions tested across studies. Although all probiotics in the studies included in the review were consumed orally by the participants, the form in which they were delivered varied. The most frequently used form of probiotics was yogurt,^34^, ^38^, ^41^, ^44^, ^45^, ^47^followed by tablet/capsule^35^, ^36^, ^39^, ^44^, ^45^, ^46^ and lozenges.^37^, ^43^ Ten studies used commercially available products,^34^, ^36^, ^37^, ^39^, ^41^, ^42^, ^43^, ^44^, ^45^ one study used their own laboratory‐developed yogurt^38^and three studies did not include information on product availability.^35^, ^40^, ^46^ The total daily dose of probiotics consumed by participants across studies ranged from 10^7^ to 2.25^11^ CFU, and the average duration of intervention was 68.3 days. The overall compliance with the probiotic intervention was moderate (49.4%–65.6%) to high (97.5%). However, five studies did not report compliance.^36^, ^41^, ^42^, ^45^, ^47^ Compliance was measured by either the percentage of people who fully complied or the average number of missing doses during the intervention phase (0.4–1.9 on average). No serious side effects were reported in any of the studies included in the review. The most commonly reported side effects were mild to moderate gastrointestinal discomfort^34^, ^35^, ^41^ and headaches^34^ that resolved spontaneously without treatments.

**TABLE 2:** Probiotic therapy characteristics (*N* = 14).

Author/Year	Form	Commercially available	Probiotic strains	Dose (CFU)	Frequency	Total duration (days)	Compliance
Guillemard/2010^34^	Yogurt	Actimel®	*Lactobacillus casei*	10^10^	Twice a day	84	97.50%
Kalima/2016^35^	Tablet	Unclear	*Lactobacillus rhamnosus* and *Bifidobacterium animalis ssp*	2 × 10^10^ and 8 × 10^9^	Twice a day	150 or 90	49.4–65.6%
Kinoshita/2019^41^	Yogurt	Meiji Probio Yogurt	*Lactobacillus delbrueckii ssp. Bulgaricus*, and *Streptococus thermophilus*	1.12 × 10^9^	Daily	112	Not reported
Kinoshita/2021^47^	Yogurt	Meiji Probio Yogurt	*Lactobacillus delbrueckii ssp*. and *Bulgaricus*	1.12 × 10^9^	Daily	112	Not reported
Kokubo/2020^42^	Yogurt	Koiwai Dairy Product	*Lactococcus lactis strain Plasma (LC‐Plasma)*	1^11^	Daily	28	Not reported
Mohammadi/2015^45^	Yogurt and tablet	ZistTakhmir	*Lactobacillus acidophilus* and *Bifidobacterium lactis* (7 strains)	10^7^	Daily	42	Not reported
Mohammadi/2016^44^	Yogurt and tablet	ZistTakhmir	*Lactobacillus acidophilus* and *Bifidobacterium lactis*	10^7^	Daily	42	90%
Pacifici/2020^36^	Tablet	Hyperbiotics PRO‐15®	*Lactobacillus spp*., *Bifidobacterium*, *and S. thermophilus (15 strains)*	225^9^	Daily	70	Not reported
Schlagenhauf/2020^37^	Lozenge	Prodentis®	*Lactobacillus reut/eri*	10^8^	Twice a day	42	83.3%
Shida/2017^38^	Yogurt	Lab‐developed	*Lactobacillus casei*	1^11^	Daily	84	99.7%
Smith‐Ryan/2019^39^	Tablet	Ecologic Barrier®	*Bifidobacterium* and *Lactobacillus acidophilus* (9 bacterial strains)	2.5 × 10^9^	Daily	42	1.9 (1) missing dose
Tubelius/2005^40^	Liquid	Unclear	*Lactobacillus reuter protectis*	10^8^	Daily	80	69.5%
Wang/2021^43^	Lozenge	Probionet®	*S. thermophilus ENT‐K12*	1^9^	Twice a day	30	“Very good”
West/2020^46^	Capsule	Unclear	*Lactobacillus acidophilus* or *Bifidobacterium animalis*	1^10^	Daily	14	0.4 (1.1) vs. 1.3 (2.5) missing dose

### 3.4 Risk of bias

As shown in Table 3, all studies had low risk of bias for appropriateness and reporting. Selection bias was minimal, with the participant assignment being completed using common and correct blinding and randomization techniques. Similarly, risk of bias for intention‐to‐treat, which is also afforded by randomization, minimizing any risk of bias that may be introduced by comparing groups that differ in prognostic variables, was moderate.^48^ Some studies were unclear in their reporting of detection bias (if outcome assessors were aware of the assigned treatment or not).^41^, ^42^, ^43^, ^47^ Lastly, there was a high risk of bias for performance (when participants or the study team are aware of the assigned treatment) and attrition (non‐random withdrawals from study groups) in all studies but one.^40^ Despite the limitations, nine out of the 14 studies had an overall low risk of bias.^34^, ^35^, ^36^, ^37^, ^38^, ^39^, ^40^, ^45^, ^46^

**TABLE 3:** Risk of Bias (*N* = 14).

Study ID	Selection bias (JBI 1,2,3)	Detection bias (JBI 5,6)	Performance bias (JBI 4,7)	Attrition bias (JBI 8)	Intent to treat (JBI 9)	Reporting bias (JBI 10,11,12)	Appropriateness (JBI 13)
Guillemard/2010^34^	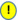	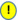	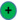	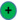	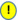	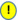	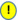
Kalima/2016^35^	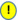	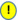	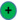	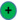	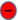	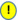	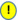
Kinoshita/2019^41^	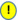	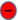	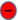	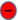	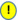	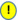	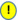
Kinoshita/2021^47^	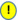	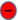	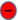	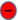	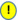	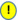	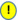
Kokubo/2020^42^	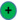	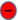	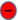	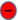	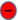	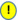	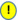
Mohammadi/2015^45^	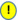	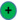	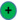	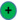	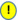	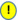	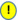
Mohammadi/2016^44^	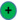	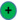	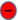	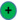	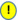	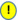	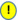
Pacifici/2020^36^	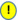	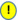	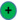	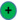	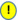	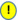	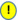
Schlagenhauf/2020^37^	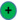	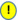	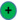	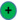	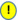	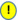	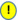
Shida/2017^38^	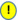	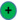	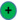	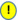	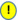	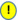	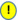
Smith‐Ryan/2019^39^	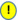	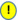	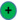	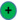	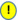	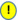	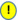
Tubelius/2005^40^	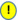	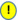	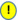	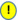	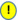	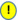	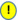
Wang/2021^43^	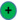	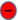	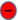	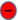	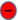	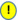	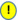
West/2020^46^	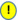	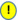	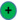	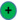	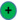	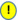	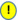

*Note*. 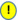, Yes; 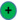, Unclear; 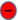, No.1. Was true randomization used for assignment of participants to treatment groups? 2. Was allocation to treatment groups concealed? 3. Were treatment groups similar at the baseline? 4. Were participants blind to treatment assignment? 5. Were those delivering treatment blind to treatment assignment? 6. Were outcomes assessors blind to treatment assignment? 7. Were treatment groups treated identically other than the intervention of interest? 8. Was follow‐up complete and if not, were differences between groups in terms of their follow‐up adequately described and analyzed? 9. Were participants analyzed in the groups to which they were randomized? 10. Were outcomes measured in the same way for treatment groups? 11. Were outcomes measured in a reliable way? 12. Was appropriate statistical analysis used? 13. Was the trial design appropriate, and any deviations from the standard RCT design (individual randomization, parallel groups) accounted for in the conduct and analysis of the trial?

*Appraisal tool used*: Appendix 3.1. JBI Critical Appraisal checklist for randomized controlled trials. Tufanaru C, Munn Z, Aromataris E, Campbell J, Hopp L. Chapter 3: Systematic reviews of effectiveness. In: Aromataris E, Munn Z (Editors)*. JBI Manual for Evidence Synthesis*. JBI, 2020. Available from: https://synthesismanual.jbi.global. https://doi.org/10.46658/JBIMES‐20‐04.

### 3.5 Probiotic outcomes in the occupational setting

As summarized in Table 4, the stress‐related health outcomes measured varied widely, but we identified four primary areas of investigation: (1) stress biomarkers, (2) common respiratory infections, (3) emotional and mental health, and (4) absenteeism/presentism. Other health outcomes that were assessed by some studies included sleep quality, gastrointestinal symptoms, cardiovascular risks, periodontal health, and metabolic composition.

**TABLE 4:** Outcomes measured in the studies by categories.

Author/Year	Measurement	Outcomes in probiotics group (compared with control group)
Biomarkers (*n* = 8)		
Guillemard/2010^34^	Hemogram, CRP, NK cells activity and count, IL‐1, 6, 8, 10, and 12, IFN‐α, β, and ˠ, TNF‐α, CCL‐5	Higher levels of leukocytes and neutrophils (*P* = .047) and NK cells (*P* < .001) No difference in levels of other WBC, CRP, NK cells, oxidative burst, or cytokines
Kinoshita/2019^41^	NK cell activity, CRP, IL‐2, 4, 5, 10, 12, and 13, IFN‐ˠ, TNF‐α, GM‐CSF	Higher levels of serum interferon gamma production (*P* = .03) No difference in levels of other inflammatory markers
Mohammadi/2015^45^	CRP, INF‐ˠ, Serum 8‐oxo‐7, 8‐dihydroguanine, protein carbonyl, 8‐iso‐PGF2a	No difference
Mohammadi/2016^44^	Serum cortisol, Kynurenine, tryptophan, neuropeptide Y, ACTH	No differences
Pacifici/2020^36^	Salivary cortisol and Ig A	Lower level of salivary cortisol No difference in immunoglobulin A
Shida/2017^38^	NK cell, salivary IgA and cortisol	Higher level of NK cell activity (*P* = .013) Lower level of salivary cortisol (*P* = .045) No difference in salivary IgA
Smith‐Ryan/2019^39^	CRP, total cholesterol, high‐density lipoproteins, leptin, glucose, adiponectin	No differences
West/2020^46^	CRP, IL‐1ra, 1β, 6, 10, IFN‐α, salivary cortisol, erythrocyte sedimentation rate, serum e‐selectin, pentraxin, MAdCAM‐1	Lower level of serum cortisol (*P* = .01), pentraxin (*P* = .001), MAdCAM‐1 (*P* = .001), and IL‐1ra (*P* = .03) during v1 to v2
Respiratory tract infections (*n* = 6)		
Guillemard/2010^34^	Self‐reported cumulated number of days with signs and symptoms	No differences in the cumulative number of diseases (the mean number of days lower in probiotic group but no statically significant) Lower level of the number of incidents in smokers (*P* = .033) Lower level of the proportion of people having s/s (*P* = .005) Delayed onset of RTI (longer time to the first s/s, *P* = .017) No difference in duration, fever, or severity of symptoms
Kalima/2016^35^	Medical visits and self‐reported symptom diary	No differences in total # of RTI symptoms, infection dx, or antibiotic prescriptions Lower episodes of in eye redness (*P* = .05), dyspnea (*P* = .04), nose symptoms (*P* = .02), and headache (*P* = .03) and # of medical visits (17 vs. 32)
Kinoshita/2019^41^	Self‐reported RTI incidence	No difference (*P* = .49)
Kokubo/2020^42^	Self‐reported daily symptom questionnaire	Lower frequency of incidents of abnormal physical condition (*P* < .01), sneezing/runny nose (*P* = .04), coughing/sore throat (*P* < .01) and lassitude (*P* = .01) No differences in fever (*P* = .35)
Shida/2017^38^	RTI characteristics as assessed by physicians	Lower cumulative incidents of RTI lower (*P* = .002) and fewer RTI s/s (*P* = .004) The URTI‐free rates were higher (0.78 vs. 0.47, CI 0.33–0.61) Time to episode is longer in probiotic group (*P* < .001) Lower cumulative number of RTI episode per person (0.3 vs. 0.7, *P* = .004) and duration of RTI episode shower (*P* = .001) No differences in severity
Wang/2021^43^	Self‐reported RTI Number of incidents Number of days with RTI s/s	Lower incidences of RTI (*P* < .005) and less days with RTI s/s (0.23 days/person vs. 1.05 days/person, *P* < .005) Shorter duration of infection episodes (2.88 days/episode vs. 4.67 days/episodes)
Emotional and mental health (*n* = 4)		
Kokubo/2020^42^	POMS Questionnaire	Higher levels of vigor (*P* = .02) No differences in anxiety, depression, anger, and fatigue
Kinoshita/2021^47^	Quality of life QOL related health SF‐8	Higher levels of general health (*P* = .02) and vitality (*P* = .01) No differences in other emotional and mental health
Mohammadi/2016^44^	DASS General health, depression, anxiety, and stress	Higher levels of general health in probiotics groups (*P* = .001) Lower level of depression and anxiety in probiotics groups (*P* = .006)
Smith‐Ryan/2019^39^	Hospital anxiety and depression scale	No difference in depression Baseline differences existed, when accounted for these differences, statistically non‐significant but clinically relevant decrease in anxiety (difference −2.3 ± 2.6) and fatigue (−4.8 ± 5.5)
Absenteeism and presentism (*n* = 3)		
Kokubo/2020^42^	World Health Organization Health and Work Performance Questionnaire	Lower level of absolute presentism
Tubelius/2005^40^	Number of sick days	Lower number of sick days (10/94 vs. 23/87, *P* < .001) Lower presentism (working while sick) 0.4 vs. 0.9 (*P* < .01) No difference in mean length of sick leave *Sub‐analysis of shift workers*: decreased absenteeism (0 vs. 9, *P* < .005)
Wang/2021^43^	Number of days lost due to respiratory tract infection	Lower number of days lost due to respiratory tract infection, (3 days (0.03 days/person) compared with 63 days (0.67 days/person), *P* < .005)
Sleep quality (*n* = 2)		
West/2020^46^	PSQI Fitbit activity tracker	Higher levels of sleep quality (*P* = .06), 22% decrease in the PSQI (improved sleep) but not significant No difference in sleep duration/time recorded on Fitbit activity Inverse correlation at baseline between PSQI and the Connor Davidson Resilience Scale (*r* = −.21, *P* = .07)
Kinoshita/2021^47^	PSQI	Higher levels of sleep quality (*P* = .007), 5.50 ➔ 5.03 vs. 5.33 ➔ 5.22
GI disturbance signs and symptoms (*n* = 2)		
Kinoshita/2021^47^	Self‐reported GI signs and symptoms	Lower frequency of constipation (*P* = .03), 1.74 ➔ 1.72 vs. 1.76 ➔ 1.84
Kalima/2016^35^	Medical visits and symptom diary	Shorter mean duration of GI symptoms decreased (vomiting, −0.7 days, *P* = .001 and stomach ache, − 0.4 days, *P* = .03)
Others (*n* = 3)		
Pacifici/2020^36^	Heart rate and systolic blood pressure	No differences
Schlagenhauf/2020^37^	Periodontal health Bleeding on probing	Higher levels in all parameters of periodontal health (*P* < .001) in situations with waning efficacy of personal oral hygiene
Smith‐Ryan/2018^39^	Body Composition and adiposity Anthropometric assessments	No difference fat mass difference (both group had decreased No difference in fat mass %, but both group decreased fat mass% by 50% in probiotic vs. 40%) No difference, both group had decreased visceral adiposity No difference in exercise performance and exercise time to exhaustion

Abbrevations: 8‐iso‐PGF2a, prostaglandin F2‐alpha; ACTH, adrenocorticotropic hormone; CRP, C‐reactive protein; GM‐CSF, Granulocyte macrophage colony‐stimulating factor; IFN, Interferon; IG, Immunoglobulin; IL, Interleukin; MAdCAM‐1, mucosal vascular addressing cell adhesion molecule 1; NK, natural killer; PC, protein carbonyl; POMS, Profile of Mood State; PSQI, Pittsburgh sleep quality index; RTI, respiratory tract infection; SF‐8, Short‐form Health survey; TNF, tumor necrosis factor; WBC, White blood cells.

#### 3.5.1 Stress biomarkers

Eight studies measured various biomarkers of inflammation and physiologic stress.^34^, ^36^, ^38^, ^39^, ^41^, ^44^, ^45^, ^46^ C‐reactive protein and natural killer cells were the most common inflammatory markers assessed (*n* = 6).^34^, ^38^, ^39^, ^41^, ^45^, ^46^ Stress markers (cortisol and immunoglobulin), pro‐inflammatory and anti‐inflammatory mediators (interleukins 1, 2, 3, 4, 6, 10, 12, and 13, interferons α, ˠ, and β, and tumor necrosis factor‐α) were also measured. Of these variables, lower levels of natural killer cells^34^, ^38^ and salivary cortisol^36^, ^38^ in the probiotic groups compared with the control groups were the only between‐group differences noted. In two studies, measures of leukocytes and neutrophils (*P* = .047)^36^ and serum interferon‐gamma production (*P* = .03)^41^ were reported to be higher in the probiotic groups compared with the control groups.

#### 3.5.2 Common respiratory tract infections

The second most frequently measured outcome was the incidents of common respiratory tract infections over the study interval (*n* = 6).^34^, ^35^, ^38^, ^41^ Respiratory infections were measured either as a self‐reported cumulative number of incidents (*n* = 5)^34^, ^35^, ^41^, ^43^ or determined by medical providers (*n* = 1)^38^ Self‐reports included the number of days experiencing clinical symptoms, which may not indicate infections, such as sneezing, coughing, or runny nose.^34^, ^41^, ^42^, ^43^ Of these six studies, the total cumulative number of respiratory tract infection in three studies were lower in the probiotic groups compared with the control groups,^38^, ^42^, ^43^ whereas the other three studies did not detect any differences between the groups.^34^, ^35^, ^41^ In addition to the total incident rates, the time to the onset of respiratory episodes was longer^34^, ^38^ and the duration of episodes was shorter^38^, ^43^ in the probiotics groups compared with the control groups. In the subgroup analysis, respiratory tract infection incidents were lower among smokers in the probiotic group.^34^

#### 3.5.3 Emotional and mental health

Four studies examined self‐rated health, fatigue, mood, anxiety, and depression. These outcomes were self‐reported using validated measurement tools, such as the Profile of Mood State,^42^ the Short‐form Health Survey,^47^ and the Hospital Anxiety and Depressions scale.^39^, ^44^ Of these, self‐rated measures of health and mood (e.g., vitality) were higher in the probiotic groups compared with the control group in two studies.^41^, ^44^ However, anxiety and depression were lower in the probiotic group compared with the control group in only one study,^44^ whereas the other three studies reported no significant differences between treatment groups.^39^, ^41^, ^43^

#### 3.5.4 Absenteeism and presentism

Absenteeism (the number of days missed) and presentism (the lost productivity that occurs when employees are not fully functioning in the workplace because of an illness) were examined in three studies.^40^, ^41^, ^43^ Both absenteeism and presentisms were consistently and significantly reduced in the probiotic groups compared with the control groups.^40^, ^42^, ^43^

#### 3.5.5 Other outcomes

Several other health outcomes were examined in some studies included in this review that could not be specifically classified under the four main areas of investigation. For example, sleep quality was measured in two studies using the Pittsburg Sleep Quality Index^46^, ^47^ and both studies reported the index to be higher (indicating better sleep quality) in the probiotics groups compared with the control groups. In two studies, signs and symptoms of gastrointestinal disturbances, such as constipation, vomiting, and stomachache, were less in the groups treated with probiotics.^35^, ^47^ Lastly, some studies measured cardiovascular risks,^36^ periodontal health,^37^ and body composition and adiposity.^39^ Interestingly, the probiotics group showed a significantly greater improvement in periodontal health compared with the placebo group (*P* < .001), while the placebo group showed a significant deterioration of all parameters (*P* < .001) at the end of the study.^37^ Other studies reported no differences in body composition^39^ or cardiovascular risks between treatment groups.^36^

## 4 DISCUSSION

The potential of probiotics to improve overall health, including mood, has led to significantly increased interest by consumers and researchers alike in recent years.^28^ Probiotics are also of interest to people who prefer non‐pharmaceutical interventions as a first‐line treatment for various stress‐related symptoms ranging from gastrointestinal distress to depression and anxiety.^16^ This scoping review aimed to examine the impact of probiotic supplements on health, stress, and stress‐related symptoms among working adults. Overall, the results of the review provide some evidence that probiotics may reduce absenteeism and presentism and improve some mental and physical health symptoms. However, the current evidence remains inconsistent due to heterogeneities in several key areas.

First, the probiotics used in the studies differed in their formula of probiotic strains, dosing, and duration of its use. For example, of the 14 studies included in the review, there were ten different formulas used. Most of the studies used a combination of sub‐species *Lactobacillus* and *Bifidobacterium* and/or *S. thermophilus*. The variety in probiotic species also led to a wide range of dosing from 10^7^ to 225^9^ CFU, and the duration for which the probiotics used in the study also varied from 14 days to 150 days. This heterogeneity creates challenges in comparing and synthesizing the interventions and their findings, thus limiting the translation and application of the findings. Even though the emerging body of science is starting to identify specific attributes among different probiotics strains, such as potential anti‐inflammatory capacity of selected Lactobacillus peptidoglycan,^49^ there are multiple mechanisms represented in different species and strains of probiotics to achieve benefits.^50^ To account for these differences in the probiotic strains and duration, we attempted to synthesize outcomes by the probiotic formula (single strain vs. multistrain) and the duration of the probiotic intervention (less than 8 weeks vs. longer than 8 weeks). However, no clear pattern emerged from the synthesis, and we were not able to draw any further conclusions.

The outcome variables across studies also varied widely. Some were subjective (e.g., self‐reported health and sleep quality), and others were objective (e.g., inflammatory markers and stress hormones). Certain subjective outcomes such as mood^42^, ^47^ self‐rated health,^44^, ^47^ and sleep quality^46^, ^47^ improved in probiotics groups, whereas others such as anxiety and depression did not differ between the probiotic and control groups. Additionally, there were no significant differences reported for the majority of inflammatory markers and stress hormones measured between treatment groups, whereas absenteeism and presentism were found to be consistently reduced in the probiotic groups, but not in the control groups.^40^, ^42^, ^43^ Nonetheless, our findings of probiotics trending towards potential benefits without conclusive evidence are echoed in other systematic reviews.^27^, ^49^ For example, Vitellio et al.^51^ and Zhang et al.^27^ examined the efficacy of probiotics on stress in healthy adults. In these reviews, the authors found that probiotic administration could generally reduce the subjective stress level of healthy volunteers and may improve their stress‐related subthreshold anxiety/depression levels. However, no significant effect was observed in the subgroup analysis, and the effects of probiotics on cortisol levels were also not significant.^27^ Another review^52^ reported that while there was a significant reduction in C‐reactive protein, TNF‐a, and other select inflammatory cytokines including IL‐6, IL‐12, and IL‐4 after probiotic supplementation, there were no reductions in IL‐1β, IL‐8, IFN‐g, or IL‐17.

These inconclusive results, however, should not be deterrents to future probiotic research. Rather, they may direct investigators in new directions. The first step towards a new direction is reframing the approaches to investigating the effects of probiotics on stress responses. The whole‐person health framework that encompasses the biological, social, behavioral, and environmental domains of a person's life should guide the design of future studies.^53^ Furthermore, a comprehensive framework is essential in evaluating probiotics studies as the consumption of probiotics and/or one's choice to consume probiotics is closely tied to the lifestyle and diet of each person. Studying one's diet, however, is essential yet notoriously tricky due to the challenges in capturing and analyzing the data.^54^ Only three studies in our review collected dietary data. While several studies^42^, ^44^, ^45^, ^46^, ^47^required a two‐ to four‐week wash‐out period before the start of the study during which probiotic‐rich diets and supplements were not allowed, no other data on diet or lifestyle factors, such as exercise, were collected.

In addition, there is also a great need for additional mechanistic investigation of probiotics through the lens of a whole‐person health framework. Probiotics are generally perceived as positive, and users of probiotics may even expect positive results.^55^ Although most of the studies in this review were randomized and blinded, the participants received a placebo, which is defined as any therapy or component of a treatment used for its non‐specific, psychological, or psychophysiological effect, but without the specific activity for the condition being treated.^56^ Aside from the provision of a specific therapeutic regimen, a placebo may still elicit non‐specific and/or contextual benefits in which the act of receiving something can potentially function as a “faux treatment” and influence outcomes.^57^, ^58^ As such, considering the potential for a placebo effect in future studies will likely result in a more comprehensive explanation of the role of probiotics in modulating stress responses in working adults.

Lastly, probiotics are largely unregulated around the world. Thus, direct and consistent evidence of their effectiveness is not required for marketing purposes. This contributes to the lack of scientific consensus on the optimal probiotic formula, dosage, and duration of therapy. Professional medical organizations, such as the American Gastroenterological Association, do not endorse probiotics due to the lack of clear evidence supporting benefits.^59^ Nonetheless, the number of adults consuming probiotics is rapidly increasing. It is currently estimated that nearly 4 million adults in the United States alone are using some form of probiotics.^60^ Therefore, a more comprehensive investigation of the psychological and physiological mechanisms of probiotics is critically needed to generate the scientific evidence required to develop treatment guidelines.

### 4.1 Limitations

To the best of the authors' knowledge, this is the first scoping review examining the effects of probiotics in occupational settings. Though we systematically reviewed the current state of the science, there are several limitations. First, we only included studies published in peer‐reviewed journals. Thus, the potential for publication bias cannot be ruled out. However, the publication bias of unintentional omission of studies with null results is not likely as several studies included in the review had null findings. Second, the occupations and work settings of the sample populations in the studies included in this review were broad. Work environments are dynamic, and there is a considerable variation in the physical and psychological stressors experienced across work settings. Nonetheless, this is the first scoping review on an emerging topic. The findings from this review can guide researchers as they design quality studies testing the effects of probiotics on workers' health.

## 5 CONCLUSION

Despite the persistent heterogeneity in critical elements across studies included in this review (e.g., content, forms, and dosages of the probiotic interventions) that limit definitive evidence, the findings that probiotics may be beneficial in improving the health of working adults are exciting for occupational health professionals. In terms of next steps, the scientific inquiry of probiotics in occupational settings requires investigations focusing on the direct and indirect mechanism of probiotics and the standardization of the strains and dosing.

## AUTHOR CONTRIBUTIONS

Jin Jun conceived the ideas; Jin Jun and Ana Kasumova searched and extracted the literature; Jin Jun, Ana Kasumova, Todd Tussing, Amy Mackos, Sheryl Justice, and Jodi McDaniel synthesized the literature, and developed and wrote the manuscript.

## FUNDING INFORMATION

Funded by the Ohio State University College of Nursing Internal Seed Grant and Faculty Start‐Up Funds (JJ).

## DISCLOSURE

*Informed consent*: N/A. *Registry*: Prospero International prospective register of systematic reviews (Registration CRD42022297808). *Conflict of interest*: The authors declare that there is no conflict of interest.

## Supporting information


Data S1.

## Data Availability

Data sharing is not applicable to this article as no datasets were generated or analyzed during conducting this scoping review. We have synthesized the current state of the literature.
