# Supplementary material for: Probiotic supplements and stress‐related occupational health outcomes: A scoping review
Source: J Occup Health. 2023 May 22;65(1):e12404. doi: 10.1002/1348-9585.12404 (PMC10203357; doi:10.1002/1348-9585.12404)
Supplement: Supplementary file 1 — Data S1. [file JOH2-65-e12404-s001.docx]

**Supplement A.** Search Terms and Search Results

1. Occupation

1. workers: "occupational groups"[MeSH Terms] OR ("occupational"[All Fields] AND "groups"[All Fields]) OR "occupational groups"[All Fields] OR "worker"[All Fields] OR "workers"[All Fields] OR "worker's"[All Fields]
2. Employee: "employee's"[All Fields] OR "occupational groups"[MeSH Terms] OR ("occupational"[All Fields] AND "groups"[All Fields]) OR "occupational groups"[All Fields] OR "employee"[All Fields] OR "employees"[All Fields]
3. Employees: "employee's"[All Fields] OR "occupational groups"[MeSH Terms] OR ("occupational"[All Fields] AND "groups"[All Fields]) OR "occupational groups"[All Fields] OR "employee"[All Fields] OR "employees"[All Fields
4. Occupational: "occupant"[All Fields] OR "occupant's"[All Fields] OR "occupants"[All Fields] OR "occupational"[All Fields] OR "occupations"[MeSH Terms] OR "occupations"[All Fields] OR "occupation"[All Fields]
5. Occupational Group: "occupational groups"[MeSH Terms] OR ("occupational"[All Fields] AND "groups"[All Fields]) OR "occupational groups"[All Fields] OR ("occupational"[All Fields] AND "group"[All Fields]) OR "occupational group"[All Fields]
6. Personnel: "occupational groups"[MeSH Terms] OR ("occupational"[All Fields] AND "groups"[All Fields]) OR "occupational groups"[All Fields] OR "personnel"[All Fields] OR "personnel's"[All Fields] OR "personnels"[All Fields]
7. Worker: "occupational groups"[MeSH Terms] OR ("occupational"[All Fields] AND "groups"[All Fields]) OR "occupational groups"[All Fields] OR "worker"[All Fields] OR "workers"[All Fields] OR "worker's"[All Fields]
8. Workers: "occupational groups"[MeSH Terms] OR ("occupational"[All Fields] AND "groups"[All Fields]) OR "occupational groups"[All Fields] OR "worker"[All Fields] OR "workers"[All Fields] OR "worker's"[All Fields]
9. vocation: "occupations"[MeSH Terms] OR "occupations"[All Fields] OR "vocation"[All Fields] OR "vocations"[All Fields] OR "vocational"[All Fields]

2. Probiotic

"Bifidobacterium"[MeSH Terms] OR "Bifidobacterium"[MeSH Terms] OR "Bifidobacterium"[Text Word] OR "Lactobacillus"[MeSH Terms] OR "Lactobacillus"[MeSH Terms] OR "Lactobacillus"[Text Word] OR "Probiotics"[MeSH Terms] OR "Probiotics"[MeSH Terms] OR "Probiotics"[Text Word] OR "Probiotics"[MeSH Terms] OR "Lactobacillus"[MeSH Terms] OR "bifidobacterin"[Supplementary Concept] OR "Probiotics"[All Fields] OR "probiotic"[All Fields] OR "microbiome's"[All Fields] OR "microbiomic"[All Fields] OR "microbiomics"[All Fields] OR "microbiota"[MeSH Terms] OR "microbiota"[All Fields] OR "microbiome"[All Fields] OR "Dietary Supplements"[MeSH Terms] OR "dietary supplement"[Text Word] OR "dietary supplementations"[Text Word] OR "food supplementations"[Text Word] OR "food supplements"[Text Word] OR "food supplement"[Text Word] OR "Nutraceuticals"[Text Word] OR "Nutraceutical"[Text Word] OR "Nutriceuticals"[Text Word] OR "Nutriceutical"[Text Word] OR "Neutraceuticals"[Text Word] OR "Neutraceutical"[Text Word]

3. Health

1. Health status

"Health"[Mesh] OR "health status"[MeSH Terms] OR ("health"[All Fields] AND "status"[All Fields]) OR "health status"[All Fields] OR ("status"[All Fields] AND "health"[All Fields]) OR "status health"[All Fields] OR "Health Status"[Mesh] OR "health"[All Fields] OR "health s"[All Fields] OR "healthful"[All Fields] OR "healthfulness"[All Fields] OR "healths"[All Fields]

1. Physical health

"physical"[All Fields] OR "physically"[All Fields] OR "physicals"[All Fields]) AND ("health"[MeSH Terms] OR "health"[All Fields] OR "health s"[All Fields] OR "healthful"[All Fields] OR "healthfulness"[All Fields] OR "healths"[All Fields])

1. Mental Health

"mental health"[MeSH Terms] OR ("mental"[All Fields] AND "health"[All Fields]) OR "mental health"[All Fields] OR Normality [tw] OR Normalities [tw] OR Normalcy [tw] OR Normalcies [tw] OR Health, Mental [tw] OR Mental Hygiene [tw] OR Hygiene, Mental [tw] OR Phenomena, Psychological [tw] OR Psychological Phenomenas [tw] OR Psychologic Processes and Principles [tw] OR Psychologic Processes [tw] OR Processes, Psychologic [tw] OR Psychological Processes [tw] OR Processes, Psychological [tw] OR Psychological Processe OR "Anxiety Disorders"[MeSH Terms] OR "Anxiety"[MeSH Terms] OR "Affect"[MeSH Terms] OR "stress"[All Fields] OR "stressed"[All Fields] OR "stresses"[All Fields] OR "stressful"[All Fields] OR "stressfulness"[All Fields] OR "stressing"[All Fields] OR "stress disorders, traumatic"[MeSH Terms] OR "Trauma and Stressor Related Disorders"[MeSH Terms] OR "stress, psychological"[MeSH Terms] OR "Physiological Phenomena"[MeSH Terms] OR "Substance-Related Disorders"[Mesh] OR "Mental Disorders"[Mesh] OR ("stress"[All Fields] OR "stressed"[All Fields] OR "stresses"[All Fields] OR "stressful"[All Fields] OR "stressfulness"[All Fields] OR "stressing"[All Fields])

1. Physiological measures (Biomarkers)

"biomarker s"[All Fields] OR "biomarkers"[MeSH Terms] OR "biomarkers"[All Fields] OR "biomarker"[All Fields] OR "Biomarkers"[Mesh] OR "Biomarkers, Tumor"[Mesh] OR "Hydrocortisone"[Mesh] OR "Neurosecretory Systems"[Mesh] OR "Pituitary-Adrenal System"[Mesh] OR "Acute-Phase Proteins"[Mesh] OR "Macrophage Inflammatory Proteins"[Mesh] OR "Systemic Inflammatory Response Syndrome"[Mesh] OR "Inflammation"[Mesh] OR "Neurogenic Inflammation"[Mesh] OR "Inflammation Mediators"[Mesh]

|  | **Query** | **Search Details** | **Results** |
| --- | --- | --- | --- |
| 5 | ((("Occupational Groups"[Mesh]) OR "Occupational Groups/organization and administration"[Mesh] OR workers) AND ("Probiotics"[Mesh] OR "Lactobacillus"[Mesh] OR "bifidobacterin" [Supplementary Concept] OR "probiotics"[All Fields] OR "probiotic"[All Fields] OR "microbiome's"[All Fields] OR "microbiomic"[All Fields] OR "microbiomics"[All Fields] OR "microbiota"[MeSH Terms] OR "microbiota"[All Fields] OR "microbiome"[All Fields] OR "Dietary Supplements"[Mesh])) AND ("biomarker s"[All Fields] OR "biomarkers"[MeSH Terms] OR "biomarkers"[All Fields] OR "biomarker"[All Fields] OR "Biomarkers"[Mesh] OR "Biomarkers, Tumor"[Mesh] OR "Hydrocortisone"[Mesh] OR "Neurosecretory Systems"[Mesh] OR "Pituitary-Adrenal System"[Mesh] OR "Acute-Phase Proteins"[Mesh] OR "Macrophage Inflammatory Proteins"[Mesh] OR "Systemic Inflammatory Response Syndrome"[Mesh] OR "Inflammation"[Mesh] OR "Neurogenic Inflammation"[Mesh] OR "Inflammation Mediators"[Mesh]) | ("Occupational Groups"[MeSH Terms] OR "occupational groups/organization and administration"[MeSH Terms] OR ("Occupational Groups"[MeSH Terms] OR ("occupational"[All Fields] AND "groups"[All Fields]) OR "Occupational Groups"[All Fields] OR "worker"[All Fields] OR "workers"[All Fields] OR "worker s"[All Fields])) AND ("Probiotics"[MeSH Terms] OR "Lactobacillus"[MeSH Terms] OR "bifidobacterin"[Supplementary Concept] OR "Probiotics"[All Fields] OR "probiotic"[All Fields] OR "microbiome's"[All Fields] OR "microbiomic"[All Fields] OR "microbiomics"[All Fields] OR "microbiota"[MeSH Terms] OR "microbiota"[All Fields] OR "microbiome"[All Fields] OR "Dietary Supplements"[MeSH Terms]) AND ("biomarker s"[All Fields] OR "Biomarkers"[MeSH Terms] OR "Biomarkers"[All Fields] OR "biomarker"[All Fields] OR "Biomarkers"[MeSH Terms] OR "biomarkers, tumor"[MeSH Terms] OR "Hydrocortisone"[MeSH Terms] OR "Neurosecretory Systems"[MeSH Terms] OR "Pituitary-Adrenal System"[MeSH Terms] OR "Acute-Phase Proteins"[MeSH Terms] OR "Macrophage Inflammatory Proteins"[MeSH Terms] OR "Systemic Inflammatory Response Syndrome"[MeSH Terms] OR "Inflammation"[MeSH Terms] OR "Neurogenic Inflammation"[MeSH Terms] OR "Inflammation Mediators"[MeSH Terms]) | 102 |
| 4 | ((("Occupational Groups"[Mesh]) OR "Occupational Groups/organization and administration"[Mesh] OR workers) AND ("Probiotics"[Mesh] OR "Lactobacillus"[Mesh] OR "bifidobacterin" [Supplementary Concept] OR "probiotics"[All Fields] OR "probiotic"[All Fields] OR "microbiome's"[All Fields] OR "microbiomic"[All Fields] OR "microbiomics"[All Fields] OR "microbiota"[MeSH Terms] OR "microbiota"[All Fields] OR "microbiome"[All Fields] OR "Dietary Supplements"[Mesh])) AND ("Anxiety Disorders"[MeSH Terms] OR "Anxiety"[MeSH Terms] OR "Affect"[MeSH Terms] OR "stress"[All Fields] OR "stressed"[All Fields] OR "stresses"[All Fields] OR "stressful"[All Fields] OR "stressfulness"[All Fields] OR "stressing"[All Fields] OR "stress disorders, traumatic"[MeSH Terms] OR "Trauma and Stressor Related Disorders"[MeSH Terms] OR "stress, psychological"[MeSH Terms] OR "Physiological Phenomena"[MeSH Terms] OR "Substance-Related Disorders"[Mesh] OR "Mental Disorders"[Mesh] OR ("stress"[All Fields] OR "stressed"[All Fields] OR "stresses"[All Fields] OR "stressful"[All Fields] OR "stressfulness"[All Fields] OR "stressing"[All Fields])) | ("Occupational Groups"[MeSH Terms] OR "occupational groups/organization and administration"[MeSH Terms] OR ("Occupational Groups"[MeSH Terms] OR ("occupational"[All Fields] AND "groups"[All Fields]) OR "Occupational Groups"[All Fields] OR "worker"[All Fields] OR "workers"[All Fields] OR "worker s"[All Fields])) AND ("Probiotics"[MeSH Terms] OR "Lactobacillus"[MeSH Terms] OR "bifidobacterin"[Supplementary Concept] OR "Probiotics"[All Fields] OR "probiotic"[All Fields] OR "microbiome's"[All Fields] OR "microbiomic"[All Fields] OR "microbiomics"[All Fields] OR "microbiota"[MeSH Terms] OR "microbiota"[All Fields] OR "microbiome"[All Fields] OR "Dietary Supplements"[MeSH Terms]) AND ("Anxiety Disorders"[MeSH Terms] OR "Anxiety"[MeSH Terms] OR "Affect"[MeSH Terms] OR "stress"[All Fields] OR "stressed"[All Fields] OR "stresses"[All Fields] OR "stressful"[All Fields] OR "stressfulness"[All Fields] OR "stressing"[All Fields] OR "stress disorders, traumatic"[MeSH Terms] OR "Trauma and Stressor Related Disorders"[MeSH Terms] OR "stress, psychological"[MeSH Terms] OR "Physiological Phenomena"[MeSH Terms] OR "Substance-Related Disorders"[MeSH Terms] OR "Mental Disorders"[MeSH Terms] OR ("stress"[All Fields] OR "stressed"[All Fields] OR "stresses"[All Fields] OR "stressful"[All Fields] OR "stressfulness"[All Fields] OR "stressing"[All Fields])) | 1,186 |
| 3 | ((("Occupational Groups"[Mesh]) OR "Occupational Groups/organization and administration"[Mesh] OR workers) AND ("Probiotics"[Mesh] OR "Lactobacillus"[Mesh] OR "bifidobacterin" [Supplementary Concept] OR "probiotics"[All Fields] OR "probiotic"[All Fields] OR "microbiome's"[All Fields] OR "microbiomic"[All Fields] OR "microbiomics"[All Fields] OR "microbiota"[MeSH Terms] OR "microbiota"[All Fields] OR "microbiome"[All Fields] OR "Dietary Supplements"[Mesh])) AND ("mental health"[MeSH Terms] OR ("mental"[All Fields] AND "health"[All Fields]) OR "mental health"[All Fields]) | ("Occupational Groups"[MeSH Terms] OR "occupational groups/organization and administration"[MeSH Terms] OR ("Occupational Groups"[MeSH Terms] OR ("occupational"[All Fields] AND "groups"[All Fields]) OR "Occupational Groups"[All Fields] OR "worker"[All Fields] OR "workers"[All Fields] OR "worker s"[All Fields])) AND ("Probiotics"[MeSH Terms] OR "Lactobacillus"[MeSH Terms] OR "bifidobacterin"[Supplementary Concept] OR "Probiotics"[All Fields] OR "probiotic"[All Fields] OR "microbiome's"[All Fields] OR "microbiomic"[All Fields] OR "microbiomics"[All Fields] OR "microbiota"[MeSH Terms] OR "microbiota"[All Fields] OR "microbiome"[All Fields] OR "Dietary Supplements"[MeSH Terms]) AND ("mental health"[MeSH Terms] OR ("mental"[All Fields] AND "health"[All Fields]) OR "mental health"[All Fields]) | 50 |
| 2 | ((("Occupational Groups"[Mesh]) OR "Occupational Groups/organization and administration"[Mesh] OR workers) AND ("Probiotics"[Mesh] OR "Lactobacillus"[Mesh] OR "bifidobacterin" [Supplementary Concept] OR "probiotics"[All Fields] OR "probiotic"[All Fields] OR "microbiome's"[All Fields] OR "microbiomic"[All Fields] OR "microbiomics"[All Fields] OR "microbiota"[MeSH Terms] OR "microbiota"[All Fields] OR "microbiome"[All Fields] OR "Dietary Supplements"[Mesh])) AND ("physical"[All Fields] OR "physically"[All Fields] OR "physicals"[All Fields]) AND ("health"[MeSH Terms] OR "health"[All Fields] OR "health s"[All Fields] OR "healthful"[All Fields] OR "healthfulness"[All Fields] OR "healths"[All Fields])) | ("Occupational Groups"[MeSH Terms] OR "occupational groups/organization and administration"[MeSH Terms] OR ("Occupational Groups"[MeSH Terms] OR ("occupational"[All Fields] AND "groups"[All Fields]) OR "Occupational Groups"[All Fields] OR "worker"[All Fields] OR "workers"[All Fields] OR "worker s"[All Fields])) AND ("Probiotics"[MeSH Terms] OR "Lactobacillus"[MeSH Terms] OR "bifidobacterin"[Supplementary Concept] OR "Probiotics"[All Fields] OR "probiotic"[All Fields] OR "microbiome's"[All Fields] OR "microbiomic"[All Fields] OR "microbiomics"[All Fields] OR "microbiota"[MeSH Terms] OR "microbiota"[All Fields] OR "microbiome"[All Fields] OR "Dietary Supplements"[MeSH Terms]) AND ("physical"[All Fields] OR "physically"[All Fields] OR "physicals"[All Fields]) AND ("health"[MeSH Terms] OR "health"[All Fields] OR "health s"[All Fields] OR "healthful"[All Fields] OR "healthfulness"[All Fields] OR "healths"[All Fields]) | 127 |
| 1 | ((("Occupational Groups"[Mesh]) OR "Occupational Groups/organization and administration"[Mesh] OR workers) AND ("Probiotics"[Mesh] OR "Lactobacillus"[Mesh] OR "bifidobacterin" [Supplementary Concept] OR "probiotics"[All Fields] OR "probiotic"[All Fields] OR "microbiome's"[All Fields] OR "microbiomic"[All Fields] OR "microbiomics"[All Fields] OR "microbiota"[MeSH Terms] OR "microbiota"[All Fields] OR "microbiome"[All Fields] OR "Dietary Supplements"[Mesh])) AND (Health status "Health"[Mesh] OR "health status"[MeSH Terms] OR ("health"[All Fields] AND "status"[All Fields]) OR "health status"[All Fields] OR ("status"[All Fields] AND "health"[All Fields]) OR "status health"[All Fields] OR "Health Status"[Mesh] OR "health"[All Fields] OR "health s"[All Fields] OR "healthful"[All Fields] OR "healthfulness"[All Fields] OR "healths"[All Fields]) | ("Occupational Groups"[MeSH Terms] OR "occupational groups/organization and administration"[MeSH Terms] OR ("Occupational Groups"[MeSH Terms] OR ("occupational"[All Fields] AND "groups"[All Fields]) OR "Occupational Groups"[All Fields] OR "worker"[All Fields] OR "workers"[All Fields] OR "worker s"[All Fields])) AND ("Probiotics"[MeSH Terms] OR "Lactobacillus"[MeSH Terms] OR "bifidobacterin"[Supplementary Concept] OR "Probiotics"[All Fields] OR "probiotic"[All Fields] OR "microbiome's"[All Fields] OR "microbiomic"[All Fields] OR "microbiomics"[All Fields] OR "microbiota"[MeSH Terms] OR "microbiota"[All Fields] OR "microbiome"[All Fields] OR "Dietary Supplements"[MeSH Terms]) AND ((("Health Status"[MeSH Terms] OR ("Health"[All Fields] AND "status"[All Fields]) OR "Health Status"[All Fields]) AND "Health"[MeSH Terms]) OR "Health Status"[MeSH Terms] OR ("Health"[All Fields] AND "status"[All Fields]) OR "Health Status"[All Fields] OR ("status"[All Fields] AND "Health"[All Fields]) OR "status health"[All Fields] OR "Health Status"[MeSH Terms] OR "Health"[All Fields] OR "health s"[All Fields] OR "healthful"[All Fields] OR "healthfulness"[All Fields] OR "healths"[All Fields]) | 1,137 |
| 0 | Clipboard | 31630991,25879690,32083132,30902791,29969291,29098783,33007981,26708682,31949878,32139154,32312311,33419325,30870486,30566363,32450813,33105353,32323706,30544607,30489352,32176565,26445629,31537512,26058758,31306390,28417309,27683185,31651219,29804245,26645377,22888972,22642690,32602753,28537898,30419498,30375452,26180243,25731901,33095208,25879811,32722609,26349657,33077817,16077253,25437604,18329134,30901440,27188901,17971827,34331994,28291486,34295504,31049751,25572586,26704712,10617996,29886377,31784669,33883746,27117843,33400303,23796758,29163938,29335554,12772433,31262754,27986469,33805289,24659567,22569299,29855372,33794969,33931660,26172395,34004448,26419583,15247120,12864796,15735075,33306561,27490209,30720778,34521465,25118085,20535553,21790446,24889392,19023818,27780238,28479217,11506826,34433082,27726925,21822678,34059794,27019455,23397688,27237824,33996997,17651958,22111592,18054688,6329920[UID] | 102 |
